# Clinical application of fecal microbiota transplantation and its influencing factors

**DOI:** 10.3389/fmicb.2026.1807071

**Published:** 2026-03-27

**Authors:** Beibei Wang, Fang Deng, Zeru Liu, Jiaxuan Tian, Yanjun Li, Yizhi Mao, Houpan Song

**Affiliations:** 1 Hunan Provincial Key Laboratory of Traditional Chinese Medicine Diagnostics, Hunan University of Chinese Medicine, Changsha, Hunan, China; 2 The Affiliated Hospital of Hunan Academy of Chinese Medicine, Changsha, Hunan, China; 3 Changsha Hospital of Traditional Chinese Medicine, Changsha, Hunan, China

**Keywords:** Alzheimer’s disease, diabetes, fecal microbiota transplantation, gastrointestinal diseases, liver disease, tumors

## Abstract

Fecal microbiota transplantation (FMT) is an emerging therapy that has received significant attention in recent years, although its origins can be traced back to 4th-century China. In modern medicine, FMT has been incorporated into clinical guidelines for the treatment of recurrent *Clostridioides difficile* infection. By re-establishing a healthy gut microbiota and regulating the immune system, FMT has potential therapeutic effects on various diseases, such as gastrointestinal diseases, diabetes, tumors, Alzheimer’s disease, and liver disease. However, its efficacy varies based on the type of disease and individual differences. The clinical application of FMT is influenced by multiple factors, including fecal matter processing, administration route, dosage, donor screening, and recipient detection. Currently, FMT faces numerous challenges, including the need to verify the stability and durability of its efficacy, standardize donor screening criteria, and optimize fecal processing and administration. Future research is expected to reveal the mechanisms of action of FMT, optimize treatment protocols, and refine its safety, efficacy, and convenience, thereby bringing hope for patients with complex and challenging diseases.

## 1 Introduction

The gut microbiota (GM) is a complex community of microorganisms, including bacteria, fungi, and viruses, that inhabits the human gastrointestinal tract. As the second genome of the human body, the GM comprises more than 98% of human microbes and contains about 10 billion cells (Rinninella et al., 2019), playing a key role in human physiological function and health. Under physiological conditions, intestinal microbes engage in complex interactions and play an essential role in maintaining intestinal balance and body health. Changes in the diversity, stability, composition, and function of the GM may lead to various diseases (Wang Y. et al., 2022). Fecal microbiota transplantation (FMT) is a therapeutic procedure involving the transplantation of fecal microbiota from a healthy donor into the recipient’s intestine, aiming to restore GM homeostasis and correct dysbiosis states, thereby enabling the treatment of microbiota-dependent diseases (Evrensel and Ceylan, 2016). It is now understood that bacteria, the dominant component of the intestinal microbiota, along with viruses, mediate the therapeutic effects of FMT by restoring bacterial homeostasis.

The earliest documented precursor to FMT dates to the 4th-century China, where the physician Hong Ge documented the use of human fecal suspension for treating toxic diarrhea in “*Zhou Hou Bei Ji Fang*” (Ge, 1956; Zhang et al., 2012). The documented method used at that time was to take the juice after grinding, yet specific procedural details have not been recorded in full. From a modern medical perspective, this approach exhibits a nascent conceptual understanding of FMT. Within the broader context of traditional Chinese medicine (TCM), further refinement of fecal derivatives (e.g., “Jinzhi,” fermented child faces buried for years, “Renzhonghuang,” fecal-fermented licorice) has been documented in ancient texts, including the “*Compendium of Materia Medica*” as therapeutic agents for febrile toxemia. These historical practices involved prolonged fermentation/burial to reduce pathogens and synergistic herb pairing, rather than direct use of raw excrement. However, modern TCM has phased out such methods due to microbiological risks and ethical concerns, retaining them solely as cultural-historical references.

In 1958, Ben Eiseman, first documented in a study (Eiseman et al., 1958) published the treatment of four patients with pseudomembranous colitis using donor stool administered via retention enema. Among them, three critically ill patients achieved significant clinical improvement. Eiseman described the outcomes as “miraculous treatments.” The study (Vrieze et al., 2012) showed that FMT is more effective than vancomycin for recurrent refractory *Clostridioides difficile* infection (rCDI) through randomized trials. The clinical practice guideline issued by the American College of Gastroenterology (Surawicz et al., 2013) became the first official clinical guideline to formally incorporate FMT into the treatment recommendation system for rCDI, while the United States Food and Drug Administration publicly established its regulatory authority over FMT for the first time, explicitly regulating feces as pharmaceutical products (Mole, 2013). In 2022, the United States Food and Drug Administration approved two microbiome-derived drugs for market use, namely Rebyota and VOWST, which are a landmark events for the recognition and application of FMT in the treatment of rCDI. Rebyota and VOWST represent live biotherapeutic products with distinct mechanisms of action. Rebyota is sourced from donor feces and is administered rectally via an enema bag (Khanna et al., 2025). In contrast, VOWST utilizes fecal microbiota spores to address rCDI through an oral, non-invasive method aimed at microbiome restoration (Blair, 2024). The emergence of non-toxigenic *Clostridioides difficile* strains and phage therapy has garnered significant attention in light of escalating antibiotic resistance. Non-toxigenic *Clostridioides difficile* strains, which lack toxin production capabilities, can colonize the gastrointestinal tract and compete with toxigenic strains by occupying the same ecological niche (Etifa et al., 2023). Phage therapy employs bacteriophages to specifically target and eliminate *Clostridioides difficile*, thereby preserving the broader GM (Umansky and Fortier, 2023). These strategies offer a more controlled and safer alternative to FMT.

Some researchers indicated that antibiotic treatment could disrupt colonization resistance. Nevertheless, the restoration of the microbiota through FMT re-establishes resistance mechanisms capable of inhibiting the growth of *Clostridium difficile* colitis (Abt et al., 2016). For instance, a study on diabetic neuropathy (Cai et al., 2018) reported that FMT treatment could enhance blood sugar control and alleviate pain symptoms. Furthermore, many studies have explored the therapeutic potential of FMT in other conditions, including urinary tract infections (Choi et al., 2023), mental illnesses (Gao et al., 2023), obesity (de Groot et al., 2017; Lee et al., 2026), and cancer (Saraiva et al., 2023). Besides, multiple factors influence the therapeutic effect of FMT. This paper discusses the factors affecting the clinical application of FMT.

## 2 Clinical application and efficacy of fecal microbiota transplantation

Gut dysbiosis is closely related to the development and functional abnormalities of the human nervous system, digestive, metabolic, and immune systems, as well as to the occurrence and progression of diseases and drug responses. While FMT has a wide range of indications, there is no unified standard for clinical efficacy evaluation. Treatment responses vary considerably across different diseases and patient populations. At present, the evaluation of the efficacy of FMT mainly focuses on symptom remission (Surawicz et al., 2013).

**Figure 1:**
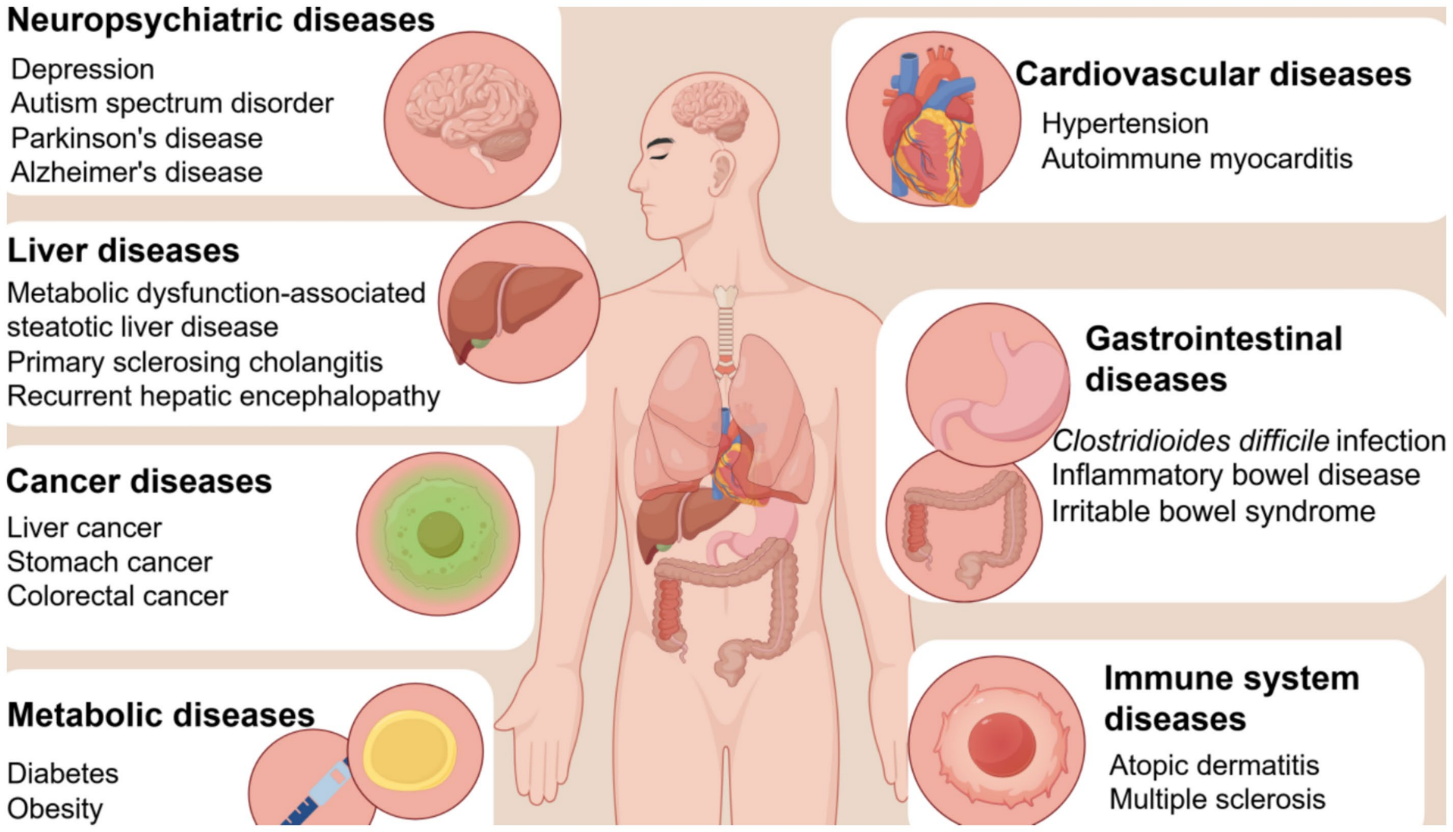
The clinical application scope of FMT. The clinical applications mainly include neuropsychiatric diseases, liver diseases, cancers, metabolic diseases, cardiovascular diseases, gastrointestinal diseases, and immune system diseases.

A growing number of trials have reported favorable therapeutic effects of FMT in gastrointestinal and extraintestinal diseases (Figure 1). In addition to rCDI, FMT can be used to treat some gastrointestinal diseases, such as inflammatory bowel disease (IBD). Regarding liver diseases, FMT has also shown certain therapeutic potential for primary sclerosing cholangitis, recurrent hepatic encephalopathy, and non-alcoholic fatty liver disease (Albhaisi et al., 2020). Moreover, FMT has shown therapeutic effects against obesity, atherosclerotic cardiovascular disease (Li Y. et al., 2026), and hypertension (Mylavarapu et al., 2026). This article mainly focuses on tumors, diabetes, IBD, Alzheimer’s disease (AD), and liver disease.

### 2.1 Tumor

The GM plays a unique role in regulating host immunity and the tumor micro-environment (TME). Regulating intestinal flora has the potential to enhance the treatment response rates in cancer patients (Hu et al., 2026). It is known that FMT enhances the anti-tumor immune response by modulating the GM (Lin et al., 2026). A higher microbial diversity, an increased production of beneficial metabolites, and the restoration of GM homeostasis contribute to improving the efficacy of immunotherapy in cancer patients (Wang M. et al., 2022). Despite extensive research efforts, there remains a lack of consensus regarding whether specific GM strains can augment the efficacy of anti-tumor immunotherapy.

In the next-generation probiotics system, *Faecalibacterium prausnitzii* and *Bacteroides fragilis*, as bioactive drug entities, have gained significant momentum. It should be emphasized that non-toxic *Bacteroides fragilis* strains can achieve a competitive protective mechanism by inhibiting the growth of *Clostridium* and *Salmonella heidelberg*, or blocking their translocation in the host, thus yielding physiological effects similar to those of traditional probiotics (Yang et al., 2024). In addition, the supplementation of a single probiotic may disrupt the balance and reduce the overall diversity of the GM. Besides, the complexity of the human GM makes it challenging to identify specific species, which hinders the cultivation of targeted strains. Current evidence suggests that GM may modulate anti-tumor immune responses and predict clinical outcomes (Śliżewska et al., 2020). Therefore, reshaping the microbiota characteristics through FMT may enhance the efficacy of host immune checkpoint inhibitors (ICIs) by modulating the immune-tumor cell interactions and altering microbial metabolites, thereby altering the TME (Liu et al., 2021; Reddy et al., 2026).

The microbiota has significant potential applications in Hepatocellular carcinoma (HCC). HCC is the most common type of primary liver cancer, accounting for approximately 90% of all primary liver cancer cases (Llovet et al., 2021). During the development and treatment of HCC, metabolic products generated by the GM enter the liver via the portal vein. These metabolites can modulate immune cells within the TME, alter the characteristics of HCC tumor cells, and influence the clinical efficacy of ICIs (Ren et al., 2025).

The microbiota has the potential to predict the body’s response to systemic treatment. *Akkermansia muciniphila*, a symbiotic bacterium colonizing the intestinal mucosal layer, has demonstrated potential in the field of probiotic applications (Zhang et al., 2019). Research (Schneider et al., 2022) has demonstrated that treatment with *A. muciniphila* in HCC animal models alleviates abnormalities, including impaired intestinal barrier function, expansion of myeloid-derived suppressor cells from monocytes, and impaired immune cell function. The enrichment of *A. muciniphila* is associated with improved survival rates in patients receiving ICI treatment. However, the use of broad-spectrum antibiotics before immunotherapy weakens the treatment effect (Peddireddi et al., 2025). Clinical studies have further confirmed that FMT or *A. muciniphila* supplements have the potential to restore patients’ responsiveness to ICI (Routy et al., 2018). Collectively, these results suggest that insufficient gut abundance of *A. muciniphila* may be linked to potential drivers of liver disease progression to HCC. Therefore, the regulation of the microbiota is expected to become a predictive biomarker and therapeutic tool for HCC.

### 2.2 Diabetes

Diabetes mellitus is a metabolic disorder caused by multiple factors. It is characterized by insulin secretion deficiency or impaired biological action, leading to persistently high blood glucose levels (Al-Jameel, 2021). Hyperglycemia can inflict chronic damage on various tissues and organs in the body, leading to dysfunction or even organ failure (Inzucchi et al., 2012). There are mainly three common types of diabetes: Type 1 diabetes (T1D), Type 2 diabetes (T2D), and other specific types of diabetes. T1D occurs more often during adolescence or childhood. T2D is the most common type of diabetes, accounting for approximately 90% of the total number of diabetes patients (Magliano et al., 2021; American Diabetes Association Professional Practice Committee, 2024).

**Figure 2:**
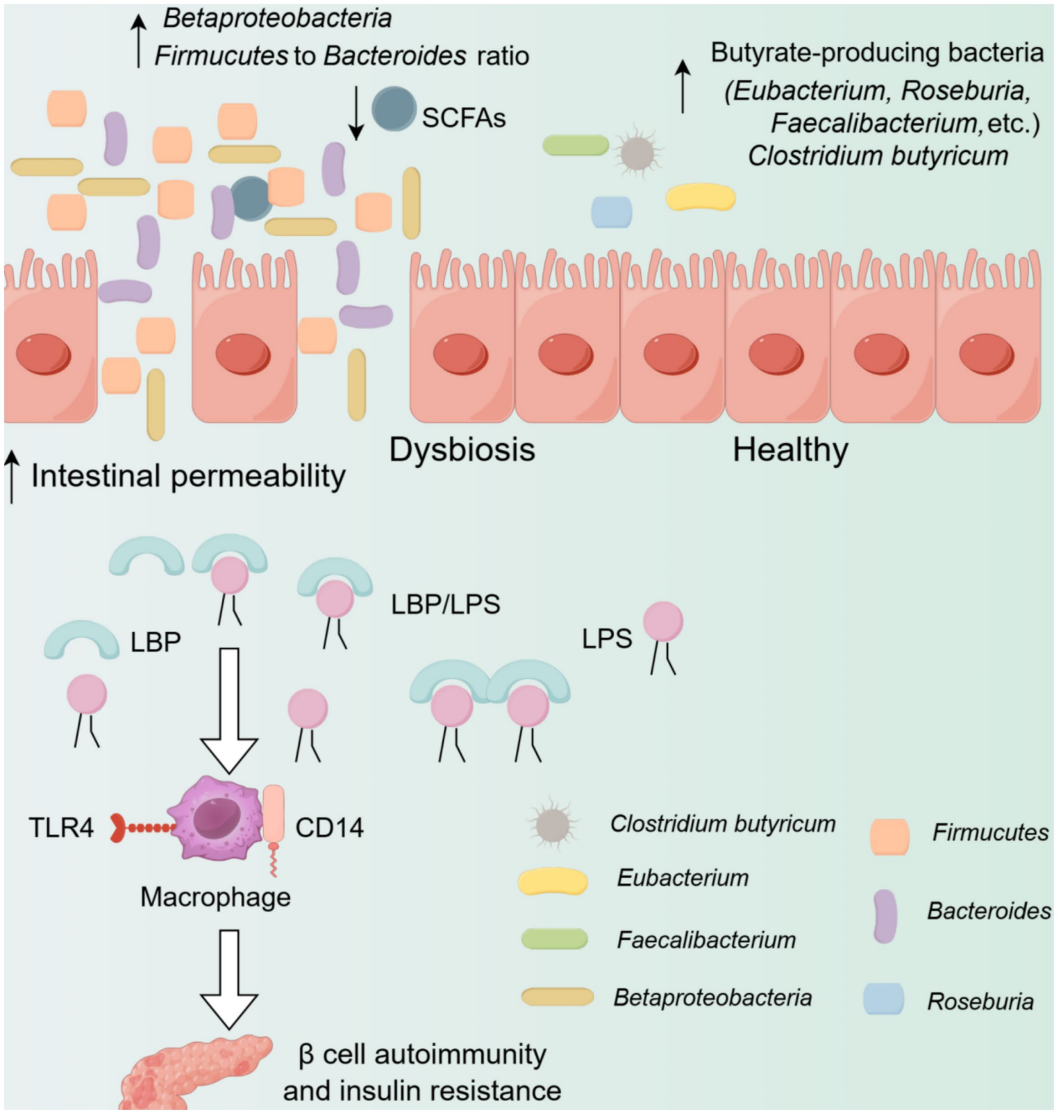
The association between gut microbiota and diabetes. On the left side, the intestinal epithelial leakage related to diabetes is shown. The ratio of *Firmicutes* to *Bacteroidetes* increases, and the number of *Betaproteobacteria* rises. LPS and form complexes with LBP, which then act on the CD14 and TLR4 receptors of macrophages, triggering signal pathway activation, leading to autoimmune response of β cells and insulin resistance. On the right side, it is the healthy intestinal epithelium. The bacteria that produce butyrate (such as *Lactobacillus rhamnosus*, *Pelegonella*, etc.) increase, and the intestinal integrity is maintained, with no bacterial translocation. LPS, Lipopolysaccharide; LBP, Lipopolysaccharide-binding protein; SCFAs, Short-chain fatty acids; CD14, Cluster of differentiation 14; TLR4, Toll-like receptor 4.

FMT can improve insulin sensitivity and alter the natural course of diabetes by regulating autoimmune mechanisms (de Groot et al., 2017). Metagenomic analyses of the GM revealed that, compared with the control group, individuals with T1D exhibited a lower abundance of butyrate-producing bacteria, as well as mucus-degrading *Prevotella* and *A. muciniphila*. Conversely, lactic-acid-producing bacteria, along with bacteria that produce short-chain fatty acids (SCFAs) other than butyrate, such as *Bacteroides* and *Ruminococcus*, were more abundant (Muscogiuri et al., 2019) (Figure 2). Butyrate, a type of SCFA, is mainly produced by certain bacterial strains. It can reduce intestinal permeability, supply nutrients to intestinal cells, and exert an epigenetic effect (Hinrichsen et al., 2021). In addition, the fibrinolytic enzyme strain has anti-inflammatory properties that can reduce the incidence of T1D in NOD mice (Mariño et al., 2017). This suggests that intestinal bacteria may play an important role in immune function disorders in patients with T1D. The study found that among patients with T2D in China, the relative abundance of *Clostridium butyricum* and its function of producing butyric acid were significantly lower than those in the normal population. However, the levels of lipopolysaccharides produced by conditionally pathogenic *Enterobacteriaceae* species, the pro-inflammatory function of hydrogen sulfide, and the function of branched-chain amino acid transport were significantly higher than those in the general population (Brunkwall and Orho-Melander, 2017; Chen et al., 2022). These changes may be related to the impaired intestinal mucosal barrier function and the increased level of intestinal inflammation in patients with T2D (Mouries et al., 2019). This indicates that intestinal bacteria may contribute to the process of immune dysfunction in patients with diabetes.

Experimental evidence suggests that FMT can enhance insulin sensitivity in patients by replenishing low-fermentation factors. This metabolic advantage is linked to the improvement of intestinal endocrine function, the modification of GM abundance, and the augmentation of donor-derived bacteria (Mocanu et al., 2021). According to the metagenomic sequencing of fecal samples, FMT can increase the colonization of beneficial bacteria in patients with T1D, and the colonization of these bacteria persists during the long-term follow-up after FMT treatment. It also has a relatively good effect on blood glucose stabilization (Weingarden et al., 2015). In a study where multiple FMTs were performed on two patients with T1D and clinical follow-ups were conducted, no adverse events were observed (Zhang et al., 2022). However, the small sample sizes in existing clinical trials limit the robustness of these findings. It is widely believed (Vatanen et al., 2018) that the therapeutic effects of FMT in treating T1D are mostly related to SCFAs. However, some studies have found that oral administration of SCFAs does not significantly improve the innate immunity or islet autoimmunity in patients with T1D. The mechanism of FMT in treating autoimmune-mediated diabetes has not been fully revealed.

### 2.3 IBD

The impaired intestinal barrier function caused by the microbiota may increase an individual’s susceptibility to IBD. IBD is a chronic inflammatory disease of the digestive tract, encompassing two main types: Crohn’s disease and ulcerative colitis (Loddenkemper, 2009). Currently, the pathogenesis has not been fully elucidated. However, studies have found significant abnormalities in the GM of patients with IBD, including a decrease in some key gut bacteria (*Faecalibacterium prausnitzii*, *Roseburia intestinalis*, *Eubacterium hallii, Eubacterium rectale*, and *Ruminococcus bromii*) and anti-inflammatory microorganisms, as well as an increase in harmful bacteria (*Ruminococcus gnavus*, *Bacteroides fragilis*, *Escherichia coli*, and *Clostridium innocuum*) (Ning et al., 2023).

**Figure 3:**
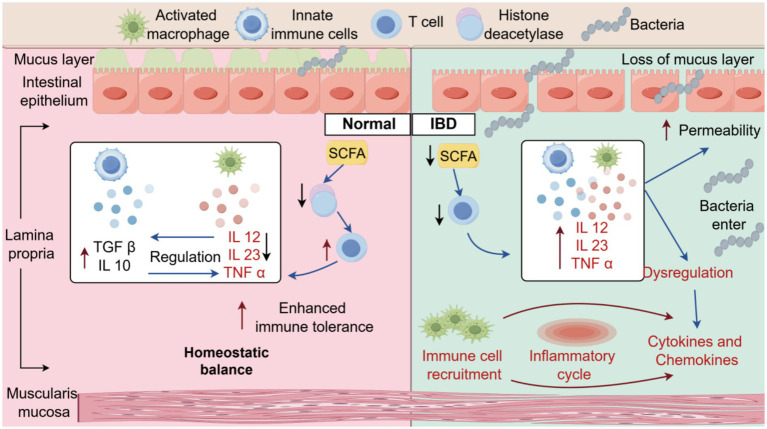
The mechanism by which microbial imbalance in the flora may exacerbate IBD. Normal condition: the mucus layer is intact. Short-chain fatty acids (SCFAs) regulate immune cells via histone deacetylase. Anti-inflammatory factors (TGF β and IL10) and pro-inflammatory factors (IL12 and TNF α) balance each other, maintaining immune tolerance and homeostatic equilibrium. IBD condition: loss of mucus layer and increased intestinal permeability allow bacterial invasion. SCFA-mediated regulation is impaired; pro-inflammatory factors (IL12, IL23, and TNF α) are overactivated, leading to immune dysregulation, immune cell recruitment, and an inflammatory cycle, which ultimately induces intestinal inflammation.

IBD development and progression are related to intestinal mucosal barrier disruption, which is caused indirectly or directly by GM dysbiosis and its metabolites (such as SCFA, bile acids, and tryptophan) (Caruso et al., 2020; Li L. et al., 2026) (Figure 3). Notably, gut dysbiosis in IBD patients disrupts the primary/secondary bile acid ratio, increasing rCDI susceptibility. Research (Ali and AlHussaini, 2026) suggests that IBD patients face a 4-fold higher rCDI risk than non-IBD individuals. FMT can restore the bile acid-metabolizing microbiota. Indeed, increasing the production of SCFA reduces intestinal permeability and inflammatory lesions, thus maintaining the integrity of epithelial cells (Bai et al., 2023). The fecal microbiota of IBD patients shows decreased expression of epithelial tight junction barrier genes and weakened tolerance of tryptophan metabolism (Wieringa et al., 2026). Therefore, FMT is recommended as a treatment option for IBD. Moreover, the combination therapy of FMT with biologics, immunosuppressants, and glucocorticoids can further increase microbial diversity and improve the therapeutic efficacy.

### 2.4 AD

AD, a neurodegenerative condition, is the leading cause of dementia, accounting for 60–80% of all dementia occurrences globally. AD is characterized by the accumulation of beta-amyloid plaques and tau-laden neurofibrillary tangles in the brain; this pathological process is closely linked to synaptic impairment, neuronal degeneration, and subsequent cognitive deterioration (Venda et al., 2010). The enteric nervous system (ENS), known as the “second brain” (Gershon, 1999), is composed of approximately 200 million neurons, making it one of the largest branches of the peripheral nervous system, regulating fundamental gastrointestinal functions to ensure microbial balance. The ENS traverses the entire length of the gastrointestinal tract and is structured into two primary ganglionic nerve plexuses: the myenteric plexus and the submucosal plexus (Sharkey and Mawe, 2023), which are present in the esophagus, stomach, and intestines. Moreover, larger mammals generally exhibit a less dense submucosal nerve plexus compared to smaller species (Spencer and Hu, 2020).

Currently, the drugs for treating AD mainly include acetylcholinesterase inhibitors and memantine (Jadhav and Kulkarni, 2023). These drugs mainly regulate the GM by targeting the brain-gut axis to inhibit neuroinflammation in the body. However, these drugs provide only short-term symptomatic relief for cognitive decline and exhibit limited ability to fundamentally halt the progression of dementia (Gupta et al., 2023).

**Figure 4:**
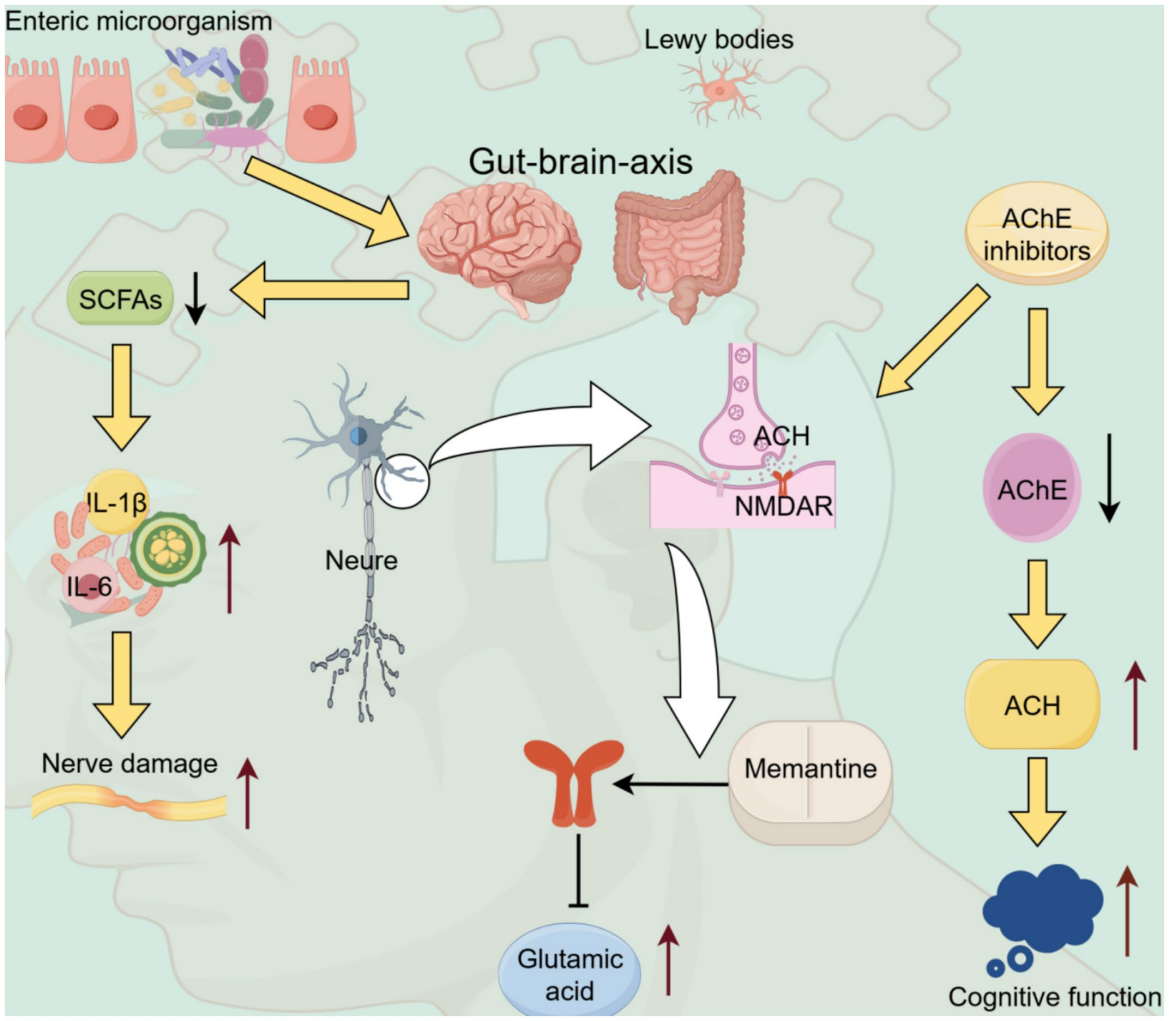
Mechanisms underlying the interaction between gut microbiota and AD. The SCFAs produced by gut microbiota can induce the production of interleukin-1β and other substances through the brain-gut axis, thereby causing nerve damage. AChE inhibitors can inhibit AChE and increase the level of ACH, thereby improving cognitive function. Memantine modulates glutamate-related pathways, exerting regulatory effects on the nervous system. The Lewy bodies may be related to certain neurodegenerative diseases. AD: Alzheimer’s disease; ACH, Acetylcholine; AChE, Acetylcholinesterase; NMDAR, *N*-methyl-D-aspartate receptor; SCFAs, Short-chain fatty acids; IL-6, Interleukin-6; IL-1β, Interleukin-1 beta.

In AD, the production and balance of metabolites from GM (such as SCFAs) may be disrupted, affecting the energy supply and metabolism of the brain, thereby accelerating the pathological progression of AD. SCFAs can regulate lipid metabolism and immune signaling. Microglia are associated with brain development (Lewandowska-Pietruszka et al., 2023). Gut dysbiosis can disrupt the production of SCFAs, trigger excessive immune responses, generate pro-inflammatory factors, and affect neurodevelopment (Srikantha and Mohajeri, 2019; Altan and Ihlamur, 2026) (Figure 4). It can also lead to lipid secretion by toxic microglia, synaptic loss, and neurodegeneration. The maturation of the GM coincides with a vulnerable period of early neurodevelopment. Thus, gut dysbiosis occurring in the early stage of life can damage the normal neurodevelopment process and become an important potential factor inducing neurodevelopmental disorders (Murciano-Brea et al., 2021; Damiani et al., 2023). Current research shows that regulating the GM represents a novel strategy for treating nervous system diseases and neurodevelopmental disorders. Its mechanism of action involves aspects such as immunity and neurotransmitter metabolism, providing new ideas and intervention directions for AD prevention and treatment (Leung and Thuret, 2015).

### 2.5 Liver disease

The disruption of the intestinal barrier function plays an important role in the development of liver diseases. With the occurrence and progression of hepatic diseases, gut dysbiosis is closely related to the changes in the function of the immune system, alterations in the composition of bile acids, and disorders in the metabolic function of the GM (Ganesan et al., 2025).

**Figure 5:**
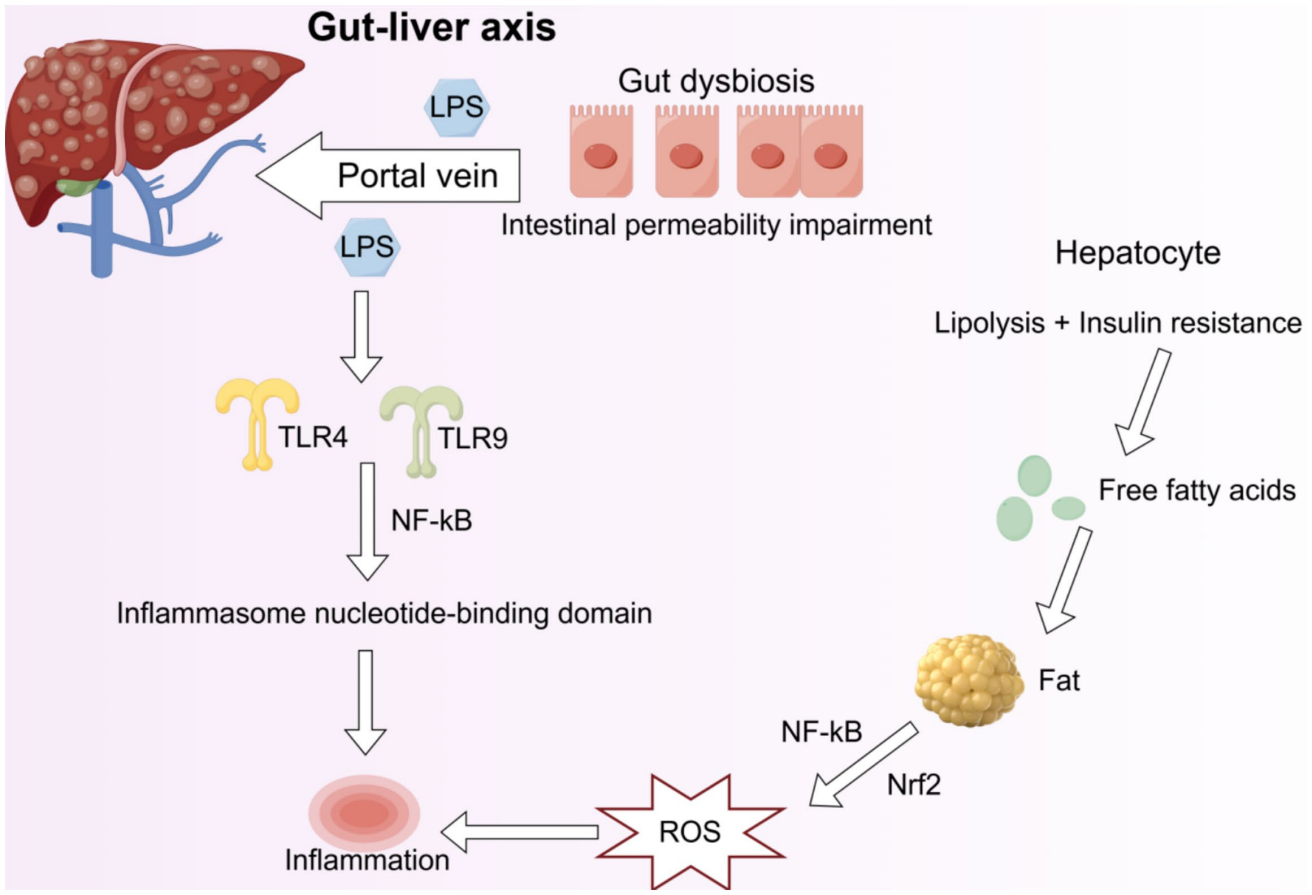
The interaction between gut microbiota and the liver. LPS enters the liver through the portal vein, activates TLR4 and TLR9, and subsequently activates the NF-κB pathway, generating the inflammasome nucleotide-binding domain, thereby causing liver inflammation. Free fatty acids enter liver cells through fat deposition, and fatty liver formation occurs due to fat production. Free fatty acids induce inflammation through the NF-κB pathway in response to oxidative stress. ROS: Reactive oxygen species; LPS: Lipopolysaccharide; NF-κB: Nuclear factor kappa-light-chain-enhancer of activated B cells; Nrf2: Nuclear factor erythroid 2-related factor 2; TLR4: Toll-like receptor 4; TLR9: Toll-like receptor 9.

Oxidative stress is closely related to the functional state of the gut-liver axis (Figure 5). It not only causes oxidative damage to DNA, lipid peroxidation, changes in the properties of oxidized proteins, and metabolic disorders of fat, and triggers inflammatory responses and tissue damage, but these factors also work synergistically to accelerate the pathological progression of liver diseases (Yang et al., 2022). Metabolically-dysfunction-associated steatotic liver disease (MASLD) has become the most common liver disease globally (Rinella et al., 2023), with an overall prevalence of approximately 37.3% (Riazi et al., 2022). Regrettably, there is still a lack of effective therapeutic approaches for this disease in clinical practice. The latest research data (Hou et al., 2025) indicate that FMT can not only significantly improve the degree of hepatic steatosis, but also regulate the biological function of innate lymphoid cells in the liver. Compared with the use of probiotics and prebiotics, FMT is more effective in achieving changes in the microbiota (Benedé-Ubieto et al., 2026). Research (Hauser et al., 2025) has shown that FMT can reverse intestinal microbiota dysbiosis, thereby impacting the developmental process of MASLD. These findings provide a solid theoretical basis and potential therapeutic strategies for the clinical application of FMT in patients with MASLD.

### 2.6 Other diseases

In addition to the disease discussed above, research (Wang P. et al., 2025) has confirmed that the GM plays a critical role in the anti-obesity effects mediated by resveratrol via antibiotic-induced microbiota depletion and FMT. Besides, resveratrol was shown to alleviate high-fat diet-related weight gain, gut dysbiosis, histopathological damage, glucose metabolism disorders, and systemic inflammation. These findings suggest that GM metabolites hold promise as potential targets for obesity prevention.

The GM also plays a significant role in the pathophysiology of depression (Fu et al., 2025). Previous research (Wang X. et al., 2025) has demonstrated that an imbalance in peripheral intestinal homeostasis contributes to depressive and anxiety symptoms during methamphetamine withdrawal, with abnormalities in intestinal flora and indole metabolism being particularly prominent. A study (Lukic et al., 2025) employed 16S rRNA gene sequencing and FMT to investigate the long-term impacts of adolescent chronic unpredictable stress on mouse behavior and intestinal flora. The results demonstrated that when the intestinal flora from mice subjected to stress during adolescence was transplanted into unstressed juvenile mice, the latter developed long-term depression-like behaviors. This finding clearly establishes that gut dysbiosis during the adolescent period plays a crucial role in the development of long-term depressive behaviors. Another study (Chen et al., 2025) revealed that either administration of lycopene (Lyc) or FMT from Lyc-treated mice could effectively alleviate Di (2-ethylhexyl) phthalate-induced anxiety and depression-like behaviors in mice. These results indicate that Lyc can exert a preventive effect against Di (2-ethylhexyl) phthalate-induced neurotoxicity through multiple pathways, including regulating the gut-brain axis, mitigating neuroinflammation, and restoring intestinal homeostasis.

Furthermore, in a study (Wang et al., 2024) using an ovalbumin-induced atopic dermatitis model established in young mice, FMT was found to regulate Th1/Th2 immune balance and intestinal flora, potentially suppressing atopic dermatitis-induced allergic reactions through the PD-L1/PD-1 pathway. A randomized, double-blind, placebo-controlled clinical trial (Liu et al., 2025) showed that patients in the FMT treatment group exhibited more significant improvements in Eczema Area and Severity Index (EASI) scores, and a higher proportion of patients achieved EASI 50 (a 50% reduction in EASI scores). No serious adverse events were documented over the trial period. These findings provide unique insights and potential new strategies for the treatment of atopic dermatitis.

The intestinal microbiota exerts a significant impact on cardiovascular health and the progression of related diseases. GM-derived metabolites, such as trimethylamine-N-oxide and SCFAs, are key mediators of the gut-heart axis (Ponce Alencastro et al., 2025). Imbalances in the GM can trigger various cardiovascular conditions, such as atherosclerosis, hypertension, and heart failure. Intervention strategies targeting the GM, including the application of probiotics, prebiotics, synbiotics, FMT, and dietary adjustments, can contribute to the regulation of blood pressure (Mahgoup, 2025). However, the differences in GM composition among individuals, coupled with insufficient data from human trials, pose challenges to the translation of these findings into clinical practice. Further research is required to determine their efficacy and long-term effects.

FMT exhibits favorable tolerability in clinical practice. Although mild to moderate intestinal irritation may occur in the peri-procedural period (Uppala et al., 2026), most adverse events are mild in severity; the most common include exacerbation of inflammatory bowel activity, diarrhea, and abdominal pain (Marcella et al., 2021). Severe adverse events are rare and are primarily attributed to patients’ underlying medical conditions (van der Sligte et al., 2026). Furthermore, inappropriate use of concomitant medications, such as antibiotics and immunosuppressants, can suppress the activity of transplanted microbiota, impair its intestinal colonization and engraftment, and thus compromise the clinical success rate of FMT.

However, several contraindications exist for FMT, suggesting it is not indicated for all patients. For instance, patients allergic to donor stool, those with severe immunodeficiency diseases, and patients in the acute infection phase are not candidates for FMT. Therefore, a comprehensive assessment of the patient’s condition is required before treatment to determine its suitability.

Currently, standardized parameters for FMT, including transplantation protocols, dosage, and timing, have not been determined. Similarly, the optimal duration of efficacy and frequency of repeated transplantations required to achieve maximal clinical benefits in patients have not been established.

## 3 Fecal processing

The processing of feces is an important part of FMT. However, due to the lack of extensive clinical research data, there is currently no standardized protocol for fecal processing. Common fecal processing methods include fresh fecal suspension, frozen fecal preparations, fecal capsules, and other similar techniques.

**Figure 6:**
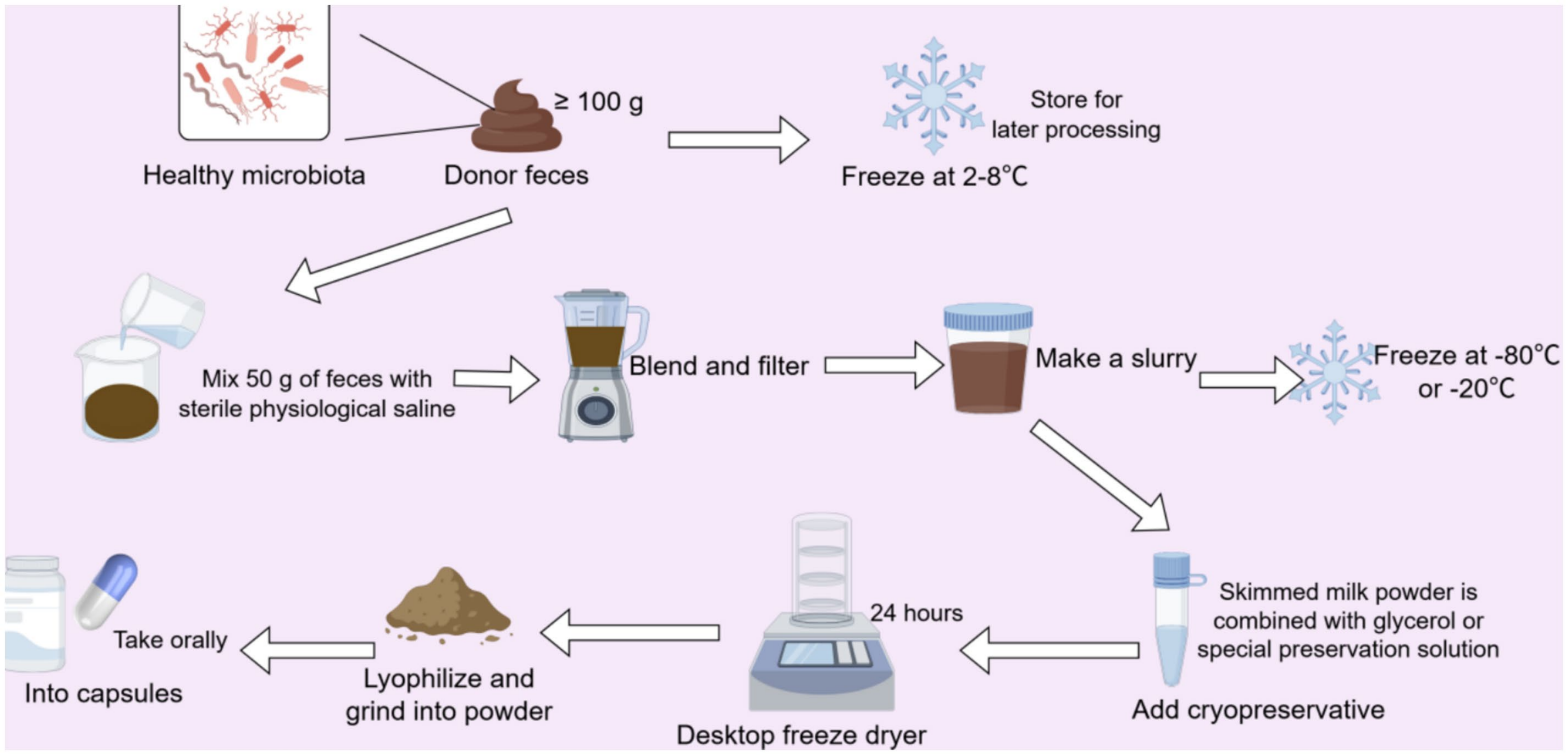
The process of preparing fecal liquid. The entire process of making an oral capsule formulation from healthy donor feces, including collection, suspension preparation, freeze-drying preservation, and freeze-drying grinding, encompasses the key steps of fecal treatment, storage, and formulation.

Donor feces are processed under aseptic conditions into a uniform fecal suspension through a series of operations, such as pulping, centrifugation, and filtration, using the fresh feces from the donor (Figure 6). Fresh fecal transplantation represents the earliest established clinical modality. It can better preserve the original state and activity of the microorganisms in the feces, which may, in principle, enhance its therapeutic efficacy. However, it is highly likely to retain some risk factors simultaneously, presenting a potential risk of infection. In addition, due to its short shelf life, it is necessary to use the fresh fecal suspension within 6 h after collection. Research has compared the clinical efficacy of frozen fecal preparation and fresh natural frozen preparation (FNFP) in the treatment of rCDI (Sintes et al., 2024), yielding clinical cure rates of 76.7 and 86.7%, respectively. Building upon the above findings, it can be concluded that FNFP has a more effective therapeutic effect than frozen fecal preparation in the treatment of rCDI.

Frozen fecal transplantation involves subjecting feces to simple processing, followed by rapid freezing at low temperatures. The feces are then thawed when needed for transplantation. This method can extend the storage time of feces and enable fecal transplantation treatment to be conducted more flexibly and conveniently. However, the freezing process may affect the activity of the microorganisms. Research (Papanicolas et al., 2019) has shown that although freezing may reduce the overall level of viable bacteria in feces, the freeze–thaw process does not significantly change the composition of the viable bacterial flora. The British Society of Gastroenterology and Healthcare Infection Society guidelines (Mullish et al., 2018) recommend banked frozen FMT material as preferable to fresh preparations for treating rCDI. In recent years, some studies have been exploring how to optimize the processing methods to improve the survival rate and activity of microorganisms, thereby enhancing the therapeutic effect of FMT.

## 4 Administration mode and dosage

FMT can be administered via several routes, broadly classified as upper gastrointestinal (including nasogastric tube, nasoduodenal tube, nasojejunal tube, and capsules) and lower gastrointestinal (such as enema, colonoscopy, and trans-endoscopic enteral tube for the colon) (Table 1).

**Table 1:** Advantages, disadvantages, and applicable scopes of various FMT routes.

FMT routes	Classification	Advantage	Disadvantage	Range of application
Upper gastrointestinal tract routes	Capsules	Non-invasive; safe and repeatable; more acceptable to patients	The microbial activity of feces may decrease; there is no reference standard; the therapeutic effect is unstable	The range is wide and it is suitable for patients with normal swallowing function
	Nasogastric tube	Simple and convenient operation; repeatable operation; can be retained; can be used for nasal feeding of nutritional liquid	The catheter is prone to come out or shift; it may cause discomfort in the nasopharynx, causing psychological problems for the patient; the fecal microorganisms may be inactivated by gastric juice	Unconditional placement of nasogastric tube; patients require repeated transplantation; those with malnutrition
	Nasoduodenal tube/nasojejunal tube	Repeatable operation; can be retained; can be used for nasal feeding of nutritional solution; can reduce gastrointestinal irritation and more effective for colonization	The catheter is prone to come out or shift; the lumen is easily blocked; it may cause discomfort in the nasopharynx; the operation is difficult; side effects	Unconditional placement of nasogastric tube; patients require repeated transplantation; those with malnutrition
Lower gastrointestinal tract routes	Enema	The operation is simple, with minimal trauma and good tolerance	The distribution of the transplantation fluid is uneven and its retention time is short; the transplantation fluid is difficult to reach the deep part of the colon	Requiring repeated transplantation; patients with lesions confined to the rectum or sigmoid colon; patients who cannot tolerate surgery
	Colonoscopy	Conductive colonoscopy examination and biopsy; the transplant fluid can precisely reach the target area	The catheter requires professional medical personnel to operate and has a high cost; it involves certain risks; and it is inconvenient to perform multiple operations	Single transplantation
	Trans-endoscopic enteral tube for the colon	Repetitive and retained	The catheter is prone to come out or shift; it may cause discomfort in the anal area	Requires multiple transplants; applicable to those with normal colon mucosa
	Sigmoid colon/proctoscopy	Feasible colonoscopy examination and biopsy; the risk is smaller	Higher risk; stronger discomfort	Patients with the lesion confined to the rectum or sigmoid colon

A nasogastric tube is used to deliver the fecal transplantation fluid into the stomach through the nasal cavity (Zhang et al., 2013). This method is relatively simple and convenient to operate, but it may cause discomfort and psychological barriers for patients. The nasoduodenal and nasojejunal tubes are used to deliver transplantation fluid into the duodenum or jejunum, reducing irritation to the stomach and intestines and ensuring more effective colonization of the microbiota. However, the nasoduodenal tube may have side effects such as the risk of reflux and intestinal irritation. Moreover, due to the relatively active peristalsis of the duodenum, the catheter is likely to be dislodged and displaced, thus affecting the effect of the transplantation. Inserting a nasojejunal tube is relatively complex. Its lumen is prone to blockage, which can affect the monitoring of the patient’s nutrient absorption and interfere with the assessment of the patient’s nutritional indicators.

An enema is a drug administration method in which fecal transplantation fluid is infused into the colon through the rectum. Although continuous FMT by fecal enema is less effective than colonoscopy-administered FMT and is associated with lower rates of sustained cure (Hota et al., 2017), it is suggested that FMT through retention enema can improve clinical status in patients who cannot tolerate surgery (Rupawala et al., 2021). This method is well tolerated and can effectively relieve rCDI symptoms in patients with multiple underlying comorbidities.

Colonoscopy involves directly introducing the fecal transplantation fluid into the colon through a colonoscope. This method can ensure that the transplantation fluid reaches the diseased area accurately, enabling it to exert a more precise therapeutic effect. It cannot be performed on patients with colonic inflammation (O’Brien et al., 2013). In addition, transendoscopic colonic intubation involves inserting a tube into the cecum through the anus to administer the transplantation fluid to the entire colon. This approach allows for multiple transplantations and has yielded promising clinical results. In a prospective observational study involving 54 patients, researchers (Peng et al., 2016) demonstrated that colonic FMT via transendoscopic enteral tubing is a novel, safe, convenient, and reliable FMT procedure, associated with a high patient satisfaction.

Oral capsules represent a non-invasive method of administration that has been developed in recent years, which is more readily accepted by patients, given that they involve taking capsules made from fecal microorganisms. Moreover, studies (Hecker et al., 2026) have shown that it exhibits good safety and effectiveness in treating certain diseases, making it more palatable to patients. However, the activity of the fecal microorganisms in the capsule formulation may be reduced. Nevertheless, randomized trials (Kao et al., 2017) have demonstrated that when the donor fecal volume is equivalent, there is no significant difference in the therapeutic effect between FMT administered via oral capsules and that administered through colonoscopy. Based on a systematic review and meta-analysis, Ramai et al. (2021) found that while colonoscopy-delivered FMT yielded a better cure rate for rCDI than enema, its efficacy was equivalent to that of capsules.

The dosage of administration is also an essential factor influencing the therapeutic effect of microbiota transplantation. The required dosage may vary depending on the specific disease and patient. Indeed, determination of the optimal dosage requires consideration of factors such as the patient’s age, body weight, and the severity of the illness. In clinical practice, doctors are required to formulate personalized administration plans according to the specific conditions of the patients.

## 5 Standardized donor screening

Most studies have focused on fecal transplantation from healthy donors. Although autologous fecal transplantation can reduce the risk of infection, improve the success rate of transplantation, and is effective for many diseases, it shows relatively lower efficacy for diseases caused by intestinal microbiota disorders. The selection of a donor is one of the essential factors affecting the success of microbiota transplantation. To ensure the safety and effectiveness of transplantation, strict standardized screening of donors is required.

The current donor screening methods mainly include questionnaires, physical examinations, and laboratory tests. The questionnaire collects information on the donor’s living habits, eating habits, family medical history, and past medical history to identify potential risk factors. The physical examination ensures donors are in good health by assessing key physiological indicators. Laboratory testing is an integral part of the screening process. It mainly involves testing the donor’s fecal samples, analyzing the microbial composition within them to ensure the activity and diversity of the microorganisms, and confirming that there are no pathogens present (Table 2).

**Table 2:** Clinical assessment of the donor.

Project		Description
	General condition	Exclusion criteria
Questionnaire	Age (Priority: 18–30 years old) Either sex BMI (18.5–24.9) In health Mental health	Donors with gastrointestinal diseases, nervous system disease, autoimmune diseases, cardiovascular disease, diabetes, metabolic syndrome, infectious diseases, and chronic pain Donors with a history of operations, additional patient history, and family medical history Donors with restrictive diet or eating disorder Donors used drugs or antibiotics within the past 3 months
Laboratory examination	Serologic examination	Infectious disease-related tests: viral hepatitis (hepatitis A, hepatitis B, and hepatitis C), HIV, EB virus, cytomegalovirus, amebiasis, and syphilis test Biochemical indicator testing: conventional biochemistry (liver function and kidney function), amylase/lipase, triglyceride, and insulin Inflammation and immune-related detection: c-reactive protein, erythrocyte sedimentation rate, and antinuclear antibody test Blood test: complete blood count (red blood cells, white blood cells, and platelets)
	Feces examination	Bacterial-related tests: detection of *Helicobacter pylori*, *Clostridium* spores, *Salmonella* bacteria, *Shigella*, *Campylobacter jejuni*, *Escherichia coli*, *Yersinia pestis*, Vancomycin-resistant *enterococci*, methicillin-resistant *Staphylococcus aureus*, and multidrug-resistant Gram-negative bacteria Virus-related tests: rotavirus, norovirus, and adenovirus Parasites and egg tests: *Ascaris* lumbricoides, *Cryptosporidium parvum*, *Ancylostoma duodenale*, *Necator americanus*, *Strongyloides stercoralis*, *Giardia lamblia*, *Entamoeba histolytica*, *Clonorchis sinensis*, and *Blastocystis hominis* Special test: occult blood test

Although donor eligibility criteria lack global standardization, they generally cover aspects such as age, health status, and living habits. The Chinese Medical Association (National Institute of Hospital Administration et al., 2022) stipulates that the age of donors should be between 18 and 30 years old (for pediatric FMT, children donors aged 3 years or above can be used under appropriate ethical approval). Eligible donors should be in good health, without a history of infectious diseases, chronic diseases, or mental illnesses. Besides, donors should maintain good lifestyle habits such as non-smoking, avoidance of excessive alcohol consumption, and a balanced diet.

Among donors, a distinct category known as the “super donor” has been documented (Moayyedi et al., 2015), which refers to individuals whose fecal matter contains high microbial diversity and an abundance of specific beneficial strains (such as *Prevotella* and *Bacteroides*). Studies have found that FMT from super donors has a higher probability of successful engraftment in recipients (Wilson et al., 2019; Li et al., 2023). Consequently, these transplants are more effective at restoring a healthy GM composition and achieving a superior therapeutic outcome.

Donors in special physiological states are individuals with distinct characteristics and conditions. For instance, athletes typically exhibit superior physical fitness and a favorable metabolic state, and their GM often differs significantly from the general population. Consequently, harnessing the feces of athletes as donors for transplantation could potentially be more efficacious in ameliorating the intestinal function and metabolic status of patients afflicted with certain metabolic disorders (Martin et al., 2025). Moreover, research has revealed that the intestines of centenarians harbor unique beneficial bacterial strains, which are likely to possess more potent antioxidant and anti-inflammatory properties. Utilizing centenarians as fecal donors could offer novel therapeutic strategies for treating aging-related diseases and preserving intestinal health.

## 6 Receptor detection

**Figure 7:**
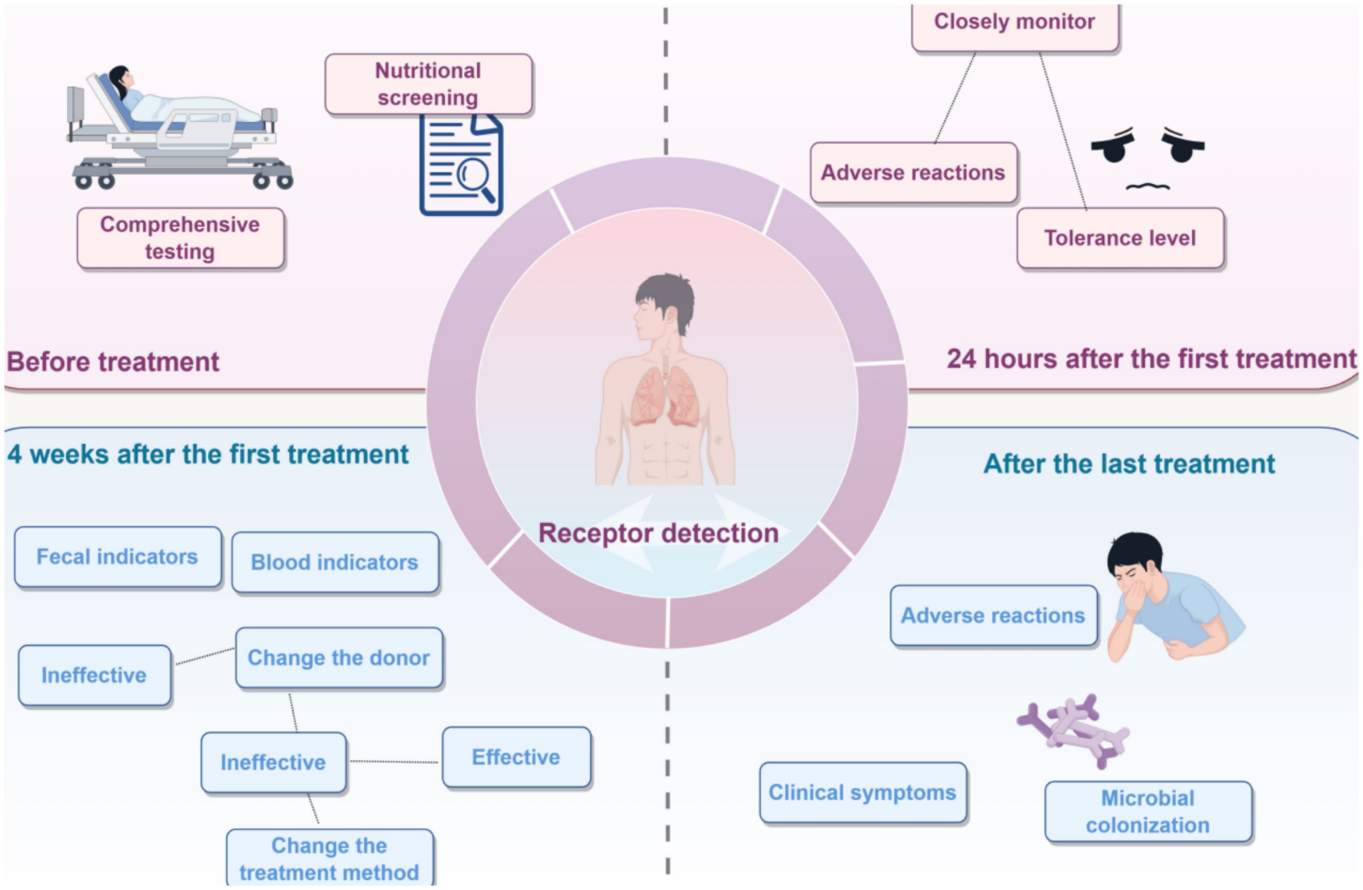
Receptor detection process. The detection and management process of the indicated receptor before treatment, 24 h after the first treatment, 4 weeks after the first treatment, and after the last treatment is described. Before treatment, a comprehensive examination and nutritional screening must be completed. Within 24 h after the first treatment, adverse reactions and tolerance should be closely monitored. Four weeks after the first treatment, fecal and blood indicators should be tested, and the donor or treatment method should be adjusted based on the therapeutic effect results. After the last treatment, attention should be paid to adverse reactions, clinical symptoms, and microbial colonization status.

Comprehensive recipient testing is required both before and after microbiota transplantation (Figure 7). Before transplantation, the main aspects to be tested include the composition of the recipient’s GM, intestinal function, and immune status. By detecting the composition of the GM, it is possible to understand the imbalance of the recipient’s gut microorganisms, providing a basis for selecting suitable donors and transplantation plans. Testing intestinal functions, such as intestinal permeability, digestion, and absorption capacity, helps to evaluate the intestinal health status of the recipient. Assessing the immune status, such as the immune cell count and the cytokine levels, can help understand the immune function of the recipient and predict the immune response after transplantation.

After transplantation, it is necessary to detect whether there are changes in the composition of the recipient’s GM and whether the donor’s microorganisms have successfully colonized. Meanwhile, clinical improvement can be evaluated through indicators, such as disease remission and enhanced quality of life. In addition, it is necessary to monitor the recipient for adverse reactions, such as infections and allergic responses. Together, these assessments enable the timely evaluation of transplantation efficacy and guide subsequent adjustments to the treatment regimen.

## 7 Conclusion

As an emerging therapeutic approach, FMT has introduced novel treatment avenues for many diseases. In this respect, it has emerged as the first-line treatment for recurrent rCDI. In the context of diabetes, FMT can improve insulin resistance by regulating the metabolic products of the intestinal flora. For IBD, it can maintain the diversity of intestinal microorganisms and protect intestinal health. FMT also regulates neuroinflammation and improves cognitive function through the gut-brain axis in AD. In liver cancer, FMT can enhance the efficacy of immune checkpoint inhibitors by reshaping the intestinal microecology and reducing chemotherapy-related intestinal toxicity. Overall, by re-establishing the balance of the GM and regulating the human immune system, FMT has great potential in treating intestinal diseases, metabolic disorders, neurodegenerative diseases, and cancer- related diseases.

FMT, as an innovative therapy targeting intestinal dysbiosis, is not only beneficial for clinical treatment, but also of great value to clinicians, patient health, and the development of the healthcare system. For clinicians, FMT focuses on the reconstruction of the intestinal microecology, enriches clinical strategies, and provides new ideas for the diagnosis and treatment of complex and refractory diseases. For patients, FMT not only improves the cure and remission rates of diseases, but also reduces the economic and psychological burdens caused by repeated medical visits and long-term medication, thereby effectively enhancing their quality of life (Wei et al., 2025). For the healthcare system, the high efficacy of FMT reduces the consumption of medical resources caused by repeated diagnosis and treatment of refractory diseases and promotes the efficient allocation of resources. Its interdisciplinary research and application drive the integration and development of microbiology, immunology, and other disciplines. Meanwhile, the clinical translation and regulatory standards of FMT also promote medical innovation and the upgrading of related industries.

Currently, there is no universal consensus on FMT in the academic community, and opinions remain highly polarized. Some researchers contend that FMT reshapes the host’s intestinal ecosystem, which may increase the risk of transmitting multidrug-resistant organisms, trigger severe infections, or even predispose individuals to certain diseases (DeLeon et al., 2025). Furthermore, its long-term effects on intestinal microecology, metabolism, and immune function remain unknown. With the exception of rCDI, research on FMT for other diseases is still at the clinical trial stage, lacks support from high-quality clinical evidence, and shows limited reproducibility. In addition, the lack of standardized protocols for donor screening, product preparation, and administration has hindered the standardized clinical application of FMT. Future studies should further elucidate the underlying mechanisms of FMT, optimize therapeutic regimens, and improve clinical efficacy. As a therapeutic approach with great potential, FMT is worthy of further in-depth research and exploration.
